# Fecal microbiota transplantation from postmenopausal osteoporosis human donors accelerated bone mass loss in mice

**DOI:** 10.3389/fcimb.2024.1488017

**Published:** 2024-12-05

**Authors:** Tinglong Chen, Ning Wang, Yongqiang Hao, Lingjie Fu

**Affiliations:** ^1^ Shanghai Key Laboratory of Orthopaedic Implants, Department of Orthopaedic Surgery, Shanghai Ninth People’s Hospital, Shanghai Jiao Tong University School of Medicine, Shanghai, China; ^2^ Shanghai Engineering Research Center of Innovative Orthopaedic Instruments and Personalized Medicine, Clinical and Translational Research Center for 3D Printing Technology, Shanghai, China

**Keywords:** osteoporosis, gut microbiota, fecal microbiota transplantation, intestinal permeability, inflammation

## Abstract

**Objectives:**

To investigate the effect of gut microbiota from postmenopausal osteoporosis patients on bone mass in mice.

**Methods:**

Fecal samples were collected from postmenopausal women with normal bone mass (Con, n=5) and postmenopausal women with osteoporosis (Op, n=5). Microbial composition was identified by shallow shotgun sequencing. Then fecal samples were transplanted into pseudo-sterile mice previously treated with antibiotics for 4 weeks. These mice were categorized into two groups: the Vehicle group (n=7) received fecal samples from individuals with normal bone mass, and the FMT group (n=7) received fecal samples from individuals with osteoporosis. After 8 weeks, bone mass, intestinal microbial composition, intestinal permeability and inflammation were assessed, followed by a correlation analysis.

**Results:**

The bone mass was significantly reduced in the FMT group. Microbiota sequencing showed that Shannon index (p < 0.05) and Simpson index (p < 0.05) were significantly increased in Op groups, and β diversity showed significant differences. the recipient mice were similar. linear discriminant analysis effect size (LEfSe) analysis of mice showed that *Halobiforma*, *Enterorhabdus*, *Alistipes*, and *Butyricimonas* were significantly enriched in the FMT group. *Lachnospiraceae* and *Oscillibacter* were significantly enriched in the Vehicle group. H&E staining of intestinal tissues showed obvious intestinal mucosal injury in mice. Intestinal immunohistochemistry showed that the expression of Claudin and ZO-1 in the intestinal tissue of the FMT group mice was decreased. The FITC-Dextran (FD-4) absorption rate and serum soluble CD14 (sCD14) content were increased in FMT mice. Correlation analysis showed that these dominant genera were significantly associated with bone metabolism and intestinal permeability, and were associated with the enrichment of specific enzymes. Serum and bone tissue inflammatory cytokines detection showed that the expression of TNF-α and IL-17A in the FMT group were significantly increased.

**Conclusion:**

Overall, our findings suggested gut microbiota from postmenopausal osteoporosis patients accelerate bone mass loss in mice. Aberrant gut microbiota might play a causal role in the process of bone mass loss mediated by inflammation after the destruction of the intestinal barrier.

## Introduction

Postmenopausal osteoporosis is a systemic bone disease caused by estrogen deficiency, characterized by decreased bone mineral density, bone microstructure changes, and increased bone fragility (Walker and Shane, 2023). Fragility fractures occur in approximately 50% of postmenopausal women (Dyer et al., 2016). Studies showed that the annual healthcare costs associated with postmenopausal osteoporotic fractures in the United States in 2023 are projected to exceed US $95 billion by 2040 (Lewiecki et al., 2019). This would put a heavy burden on the health of middle-aged and elderly women and the medical expenditure of society.

Previous studies demonstrated the critical influence of the gut-bone axis on bone metabolism (Guss et al., 2017; Villa et al., 2017; Tyagi et al., 2018). A direct relationship between gut microbiota and bone mineral density had been reported (Sjögren et al., 2012; Chevalier et al., 2020). Intestinal immune status is an important cause of bone loss in estrogen deficiency (Li et al., 2016). Therefore, it is of great significance to study the regulation of gut microbiota on bone mass.

Fecal microbiota transplantation (FMT) is a promising strategy to modulate gut microbiota (Kelly, 2013). Transfer gut microbiota of hypertensive patients into germ-free mice observed elevated blood pressure characterized by microbial transfer (Li et al., 2017). A depressive state could be transferred to germ-free mice via gut microbiota from patients with major depressive disorder (Zheng et al., 2016). In addition, manifestations of liver disease, obesity, type 2 diabetes mellitus, and ischemic stroke have also been reported to be transferred to mice by gut microbiota (Fei and Zhao, 2013; Le Roy et al., 2013; Benakis et al., 2016; Llopis et al., 2016; Gurung et al., 2020). However, whether osteoporosis patient characterization could be transferred by gut microbiota has not been reported.

Here, we first transplanted the gut microbiota from postmenopausal osteoporosis patients into mice to investigate the role of gut microbiota in bone regulation. We found that postmenopausal osteoporosis bone mass loss characterization was transferred to ABX mice, which exhibited gut microbiota alterations and gut barrier impairment.

## Methods

### Subject characteristics and sample collection

Fecal samples were collected from postmenopausal women, and all experimental procedures were ethically approved by the Medical Ethics Committee of Shanghai Ninth People’s Hospital (SH9H-2019-T101-2). Participants were recruited from the Osteoporosis Clinic of Shanghai Ninth People’s Hospital. Informed written consent was obtained from all participants, adhering to the Declaration of Helsinki.

All the subjects in the current work were strictly enrolled. They were from a relatively concentrated environment, the differences in the diets were relatively small. Inclusion criteria for postmenopausal women were as follows: age older than 50 years and at least 12 months since their last menstrual period. Exclusion criteria encompassed cancer, chronic liver disease, heart disease, renal disease, diabetes, and secondary osteoporosis-related conditions (hyperthyroidism, steroid abuse, Cushing’s syndrome, hyperparathyroidism, etc.). The participants using antibiotics or affected bone metabolism drugs (such as estrogen and glucocorticoids) over the past three months, or are currently were excluded. Unclear primary osteoporosis diagnosis, untreated osteoporosis, or a history of brittle fracture of individuals are excluded. Besides, factors that might affect the gut microbiome were taken into account. Participants taking probiotics were excluded. After the age and weight matching. Finally, five postmenopausal osteoporosis women and five healthy controls were included.

Dual-energy X-ray absorptiometry (DXA, Hologic Discovery A, United States) was then utilized to measure bone mineral density (BMD) and T-score in the postmenopausal women donors. The two groups were defined based on the T-score threshold corresponding to BMD. Following the World Health Organization’s definition of osteoporosis, a T score of ≤ -2.5 indicates osteoporosis, while a T score of ≥ -1 indicates normal bone mass (Kanis, 1994; Compston et al., 2019). Subjects were categorized into the postmenopausal osteoporosis group (n=5) and the healthy control group with normal bone mass (n=5) as donors for fecal microbiota transplantation.

Fresh stool samples were collected from each participant within 15 minutes of excretion, then promptly frozen and preserved in liquid nitrogen for subsequent fecal microbiota transplantation.

### Experimental animals

Fourteen 7-week-old female C57BL/6 mice were procured from Shanghai Jiesijie Laboratory Animal Co., LTD. The mice were housed in a specific pathogen-free (SPF) environment with a 12-hour light-dark cycle, and they had ad libitum access to pelleted rodent feed and autoclaved water. After one week of acclimation, the mice were randomly assigned to two groups: the Vehicle group (n=7) received intestinal bacteria transplantation from the healthy control group, and the FMT group (n=7) received intestinal bacteria transplantation from postmenopausal osteoporosis patients. All animal experiments were ethically approved by the Ethical Committee of the Ninth People’s Hospital of Shanghai Jiao Tong University School of Medicine and adhered to the recommendations of the Guide for the Care and Use of Laboratory Animals by the Chinese Association for Laboratory Animal Science.

### Antibiotic cocktail treatment

Based on previous studies, both groups of mice were treated with an antibiotic cocktail (ABX) to establish a pseudo-sterile mouse model (Borody et al., 2013; Liu et al., 2020; Wang et al., 2020). C57BL/6 mice received a mixture of antibiotics (Vancomycin, 100 mg/kg; Neomycin sulfate 200 mg/kg; Metronidazole 200 mg/kg; Ampicillin 200 mg/kg) via gavage for 14 days, with the drinking solution changed every other day to deplete the gut microbiota. Fresh fecal samples were collected on day 15.

### Fecal microbiota transplantation

Fecal microbiota transplantation (FMT) was conducted following established methods (Hamilton et al., 2012; Borody et al., 2013). The feces (1.5 g) of five humans collected from each group were mixed, steeped, and shaken in sterile PBS (pH 7.4, 10 mL) and then filtered through a 100 μm pore mesh. Centrifugation at 800G for 3 minutes was performed to obtain two sets of supernatants, which were subsequently frozen at −80°C. At 8 weeks of age in the pseudo-sterile mice, the diluted supernatant (200μL per mouse) was administered to recipient mice daily via gavage for 8 weeks. The body weight of the two groups of mice was monitored weekly. One day before euthanasia, the mice were fasted for 12 hours, and fecal samples were collected and stored at −80°C. Then, the mice were weighed after anesthesia. And femur, serum, and colon tissues were collected from each mouse for subsequent analysis. The operation flow chart was created using Adobe Illustrator 2020 (V24.0.1.341).

### Serum, intestinal, and bone tissues collection

At the end of the experiment, mice were fasted for 12 h and then given sodium pentobarbital (100 mg/kg). Mice were sacrificed (Euthanasia by CO_2_ inhalation) after blood collection from the abdominal aorta. Serum was obtained by centrifuging the blood for 10 min at 4000 rpm/min (4°C). Bilateral femoris were dissected, removed, and fixed in 4% paraformaldehyde. Approximately 2 cm of small intestine was removed and washed with PBS. A portion of the small intestine was frozen at − 80 °C and the remainder was fixed in 4% paraformaldehyde, for subsequent analyses.

### Bone mineral density measurement and micro-CT analysis

Ipsilateral femur samples from each group were collected and fixed in 4% paraformaldehyde. The bone structure of mouse femurs was evaluated using a high-resolution CT81 system (Scanco Medical AG, Bruettiselien, Switzerland) (Fu et al., 2020). The structural parameters of the femur were further analyzed using program CTAn (Bruker, Karlsruhe, Germany). 3D reconstruction of the tibial metaphyseal was performed using the built-in Scanco software.

### Enzyme-linked immunosorbent assay

Mice serums were separated by centrifugation (2500 × g, 10 min) and stored at – 80°C. CTX-1 elisa assay kits (LSBio cat. no. LS-F21349), Soluble Cluster of Differentiation 14 (sCD14) elisa assay kits (Cell Sciences cat. #: CKM034), TNF-α elisa assay kits (Beyotime, China. cat. no.PT512), IL-17A elisa assay kits (Beyotime, China. cat. no. PI545) were used to ELISA measured. All procedures were performed according to the manufacturer’s instructions.

### Measurement of fasting blood glucose content

Fasting blood glucose from mice was measured with the use of a handheld glucose meter, FreeStyle Lite Glucose strip (Abbott Laboratories), from serum samples obtained by centrifugation after a 12-hours fast.

### *In vivo* intestinal permeability measurements

After fasting for 4 h, mice were given 200 μl FITC-dextran (FD4) (440 mg/kg body weight) by gavage, and blood samples were collected 4 h later. The concentration of FITC-dextran was determined using a fluorometer with an excitation wavelength of 490nm and an emission wavelength of 530 nm. Fitc-dextran in serum was serially diluted. It was used to establish a standard curve.

### DNA extraction, amplification, and shallow shotgun sequencing

Microbial DNA was extracted from the stool samples using the QIAamp DNA Stool Mini Kit (QIAGEN, USA). The DNA was subsequently fragmented into 400 bp readings and processed with the Biomek^®^ FXp (Bio Scientific, USA) compatible NEXTflex™ DNA sequencing kit to construct a paired-end genomic library. Paired-end reads were generated using an Illumina HiseqTM2500 platform. Raw FASTQ files were filtered using the FASTX-Toolkit. The high-quality reads were then subjected to splicing and assembly using Mothur software (V1.44.1) with the main splicing parameter Kmer values set between 55 and 85. Scaffolds exceeding 500 bp were selected for bioinformatics analysis in preparation for shallow shotgun sequencing.

Shallow shotgun sequencing was conducted using the Illumina NovaSeq sequencing platform. Predicted gene sequences in the stool samples were clustered using CD-HIT (version 4.6.1) with parameters set to 90% identity and 90% coverage to construct non-redundant gene sets. The high-quality readings of each sample were then aligned with the non-redundant gene set (95% identity) using SOAPaligner software (version 2.21), and the abundance of genes in each sample was calculated. Sequences of the non-redundant gene sets were compared with the database RefSeq version 82 using Diamond (version 0.8.35) with an expected value of 10^-5^ for blastp alignment. Species annotations were obtained through the corresponding taxonomic information database of the database RefSeq, and species abundances were determined by summing the abundances of the corresponding genes for each species (Wang et al., 2022b).

### Bioinformatic analysis

Alpha diversity analysis (ACE, Chao, Shannon, and Simpson indices) were conducted using Mothur software (V1.44.1). Principal component analysis (PCOA) was employed to visualize the similarities or differences between the two groups by calculating ecological distance. LEfSe (linear discriminant analysis effect size) was utilized to compare the two groups, and the Kruskal-Wallis test and paired Wilcoxon rank-sum test and linear discriminant analysis (LDA) were employed to identify biomarkers with both statistical differences and biological significance, aiming to identify features with different abundances and related categories. The enzyme functions were classified based on the NC-IUBMB classification and nomenclature of enzymes (commonly known as the Enzyme List, an officially recognized functional classification system). Enzyme data can be found in IntEnz (http://www.ebi.ac.uk/intenz), supported by NC-IUBMB (Fleischmann et al., 2004). Enzyme abundances between groups were compared by Benjamini-Hochberg FDR. Corrected p < 0.05 was considered significant in predicting pathway status across the community (Hillmann et al., 2018).

The α diversity visualization, columnar stacking maps at the phylum, genus, and species levels, principal coordinate analysis, and correlation heat map were generated using R software (V4.2.2). Enzyme enrichment analysis visualization was performed using stamp (V2.1.1.0). Welch’s t-test and White’s non-parametric t-test were used to compare profiles between the two groups, and the effect size was measured using the difference in mean proportion along with Welch’s confidence intervals. Principal component analysis (PCA) plots and extended error bar plots were created based on corrected p-values (Parks et al., 2014). LEfse analysis and linear discriminant analysis visualization by Galaxy cloud Platform implementation (galaxy Platform, http://huttenhower.sph.harvard.edu/galaxy) (Boekel et al., 2015).

### Femoral and ileum section staining

Bilateral femoral samples were collected and fixed in 4% paraformaldehyde. Decalcification was performed with a decalcification solution containing EDTA for 2 weeks at room temperature. After decalcification, the femoral tissue was embedded in paraffin and cut longitudinally into 5 μm thick sections. The sections were subjected to H&E (Hematoxylin-eosin) and TRAP (Tartrate resistant acid phosphatase) staining using the TRAP staining kit (Servicebio, Wuhan, China). After washing off the staining solution, the HE-stained and TRAP-stained femur sections were observed under an optical inverted microscope (Olympus, Japan), and the number of TRAP-positive osteoclasts was quantified using ImageJ software (NIH, Bethesda, MD, USA).

After fixation with 4% (v/v) paraformaldehyde, the distal ileum of mice was embedded in paraffin and cut into 4 µm thick sections. The sections were stained with H&E to observe the morphological features of the two groups under an Olympus light microscope (Japan).

### Immunohistochemistry and immunofluorescence staining

After blocking sections with 0.3% triton X-100, in Tris-hcl buffer (0.1 M) containing 5% fetal bovine serum, Incubation with anti-ZO-1 (1:100, NOVUS), anti-claudin (1:100, SAB signaling pathway antibody), and anti-occludin (1:100, Proteinbtech) primary antibodies was performed overnight at 4 °C. Jackson Immunology Research Laboratories LTD), secondary antibody was FITC-labeled goat anti-rabbit and Fluor 488-conjugated goat anti-mouse (GeneTex, Irvine, CA, USA). Nuclei were counterstained with 40, 6-diamidino-2-phenylindole (DAPI) (1:30 000 Roche Biochemistry, Monza, IT). A laser scanning confocal microscope (Olympus, Japan) was used for image acquisition. ImageJ software was used for quantification (NIH, Bethesda, Maryland, USA).

For immunofluorescence of femora, the primary antibodies were anti-TNF-α (Servicebio, China) and IL-17A (Servicebio, China), the secondary antibodies were Cy3-labeled goat anti-mouse IgG (Servicebio, China), Fluorescence intensity quantification was done using Image J. The remaining procedures were the same as above.

For OCN staining, femur sections were antigen repaired and then blocked with 100%BSA and Triton 3. The sections were then treated with primary anti-osteocalcin antibodies (catalog M173; Takara) at 4°C overnight. Immunoreactivity was determined using HRP-DAB cells in the tissue staining kit according to the manufacturer’s instructions (Yang et al., 2019).

### Statistical analysis

GraphPad Prism 9.0 statistical software was used to visualize body weight gain, all skeletal parameters, and count data of osteoblasts and osteoclasts in mice. SPSS (SPSS^®^Statistics v26) was used for independent-samples T test to analyze statistical differences. The overall quantified data were presented as mean ± standard deviation (SE). P-value < 0.05 was considered statistically significant.

## Results

### Basic information on donor population and recipient mice

Ten female subjects provided fecal samples, including 5 postmenopausal women without osteoporosis as healthy controls (Con group) and 5 postmenopausal women diagnosed with osteoporosis (Op group). The inclusion and exclusion criteria were detailed in the Methods section. The age and body weight of the two groups were comparable (P > 0.05). The mean bone mineral density (BMD) was significantly lower in the Op group (P < 0.05) (**Table 1**).

**Table 1:** Human donor demographics.

Demographics	Healthy(n=5)	Postmenopausal osteoporosis (n=5)	p-value
Gender (F/M)	5/0	5/0	1
Age (y)	60.4 ± 2.97	61.2 ± 4.15	0.242
Weight (kg)	58.6 ± 3.51	58.5 ± 5.45	0.973
BMI (kg/m^2^)	23.08 ± 3.11	21.02 ± 1.95	0.330
BMD	0.95 ± 0.04	0.71 ± 0.13	0.004
T-score	-0.64 ± 0.26	-3.56 ± 0.73	<0.001

Independent-samples t-test was used to compare continuous variables. Measurement data were expressed as mean ± standard deviation.

**Figure 1:**
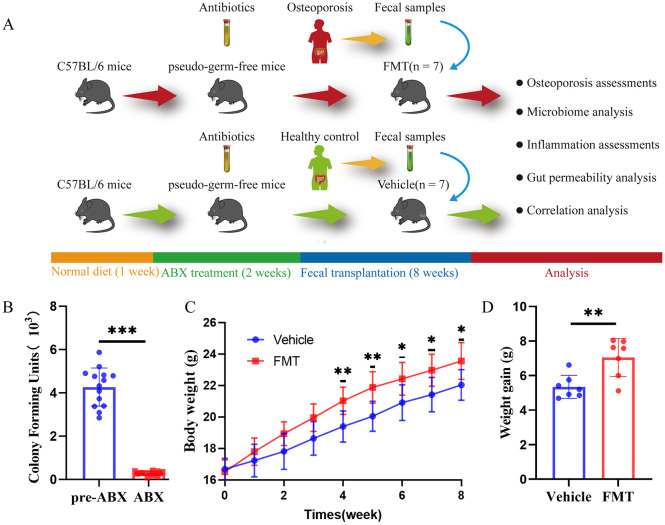
Operation flow chart and basic information in mice. **(A)** Overall schematic representation of fecal microbiota transplantation. C57BL/6J mice were acclimated for 1 week followed by oral administration of antibiotics for 2 weeks followed by oral inoculation with prepared fecal contents from two different populations (Con, Op). After successful colonization of the gut microbiome, feces, bone tissues, intestinal tissues, and inflammation were collected for further analysis. **(B)** Colony counts of mice before and after ABX treatment showed significant differences before and after antibiotic treatment in mice. **(C)** Weight growth curves of mice in each group. **(D)** The weight gain of mice in both groups, and the difference between the two groups was significant, * stands for P<0.05, ** stands for P<0.01, and *** stands for P<0.001. **(B–D)** Independent-Samples t-test.

The FMT process was depicted in **Figure 1A**. Colony counts before and after ABX treatment indicated a significant impact of antibiotic administration before FMT, successfully establishing a pseudo-sterile mouse model (**Figure 1B**). The body weight of the mice during FMT was recorded. The FMT group showed faster weight gain after receiving the gut microbiota of Op patients, showing a statistically significant difference from 4 weeks after receiving gut microbiota transplantation (p < 0.05, **Figure 1C**). After 8 weeks, compared with the weight before fecal microbiota transplantation, the weight gain of the mice in the FMT group was significantly higher than that in the Vehicle group (p < 0.01, **Figure 1D**).

### Mice received gut microbiota from osteoporosis patients had a significant reduction in bone mass

**Figure 2:**
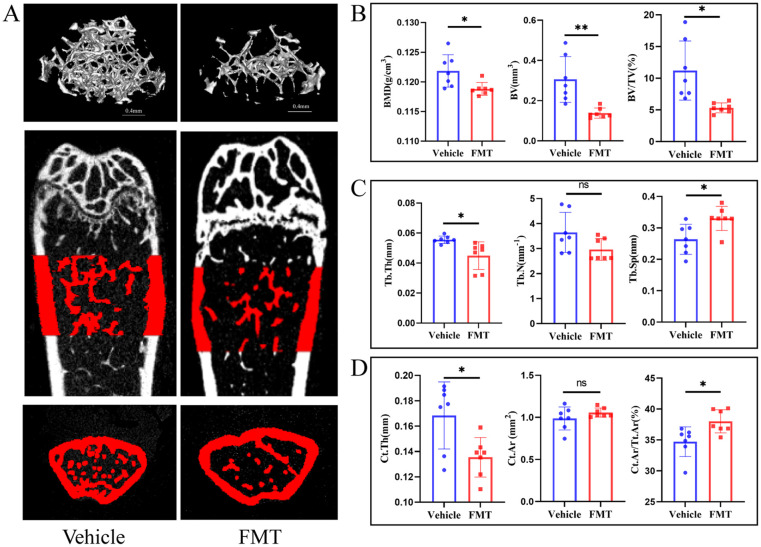
Micro-CT and bone tissue parameters in mice. **(A)** Micro-CT image of the trabecular bone structure of the distal femur. The scale bar represents 0.4mm. **(B)** Common bone parameters of mouse femurs. Including bone mineral density (BMD), bone volume (BV), and bone volume fraction (BV/TV). **(C)** Trabecular bone parameters of the femur in mice. Including the number of trabecular bone (Tb. N), the average thickness of trabecular bone (Tb. Th), and trabecular bone separation (Tb. Sp). **(D)** Cortical bone parameters. Cortical bone thickness (Ct. Th), cortical bone area (Ct. Ar), and the ratio of cortical bone area to total bone area (Ct. Ar/Tt. Ar). * stands for P<0.05, and ** stands for P<0.01. **(B-D)** Independent-Samples t-test.

We first analyzed mouse bone tissue to verify the transfer of osteoporosis representations. Micro-CT analysis was conducted to assess changes in the trabecular bone structure of the distal femur in mice inoculated with the intestinal flora of postmenopausal osteoporosis patients. Three-dimensional images (**Figure 2A**) revealed evident bone loss in the FMT group, with an enlarged medullary cavity and reduced bone trabeculae in the distal femur. Furthermore, the common bone parameters showed that the FMT group had significantly lower bone mineral density (BMD, p < 0.05), bone volume (BV, p < 0.01), and bone volume fraction (BV/TV, p < 0.05). (**Figure 2B**). Then, we analyzed bone microstructural parameters to assess bone condition. FMT group had less trabecular number (Tb. N, p > 0.05), smaller trabecular thickness (Tb. Th, p < 0.05), and higher trabecular separation (Tb. Sp, p < 0.05) (**Figure 2C**). To obtain a more comprehensive understanding of the bone condition during fecal microbiota transplantation, the related parameters of cortical bone were analyzed. The results showed that cortical bone thickness (Ct. Th, p < 0.05) was significantly decreased in the FMT group. There was no significant difference in cortical bone area (Ct. Ar, p > 0.05) between the two groups. However, the ratio of cortical bone area and total cortical bone area (Ct. Ar/Tt. Ar, p < 0.05) was significantly higher in the FMT group (**Figure 2D**). These results indicate that the bone loss and bone microstructural changes in the FMT group of mice, which were similar to those in osteoporosis patients.

**Figure 3:**
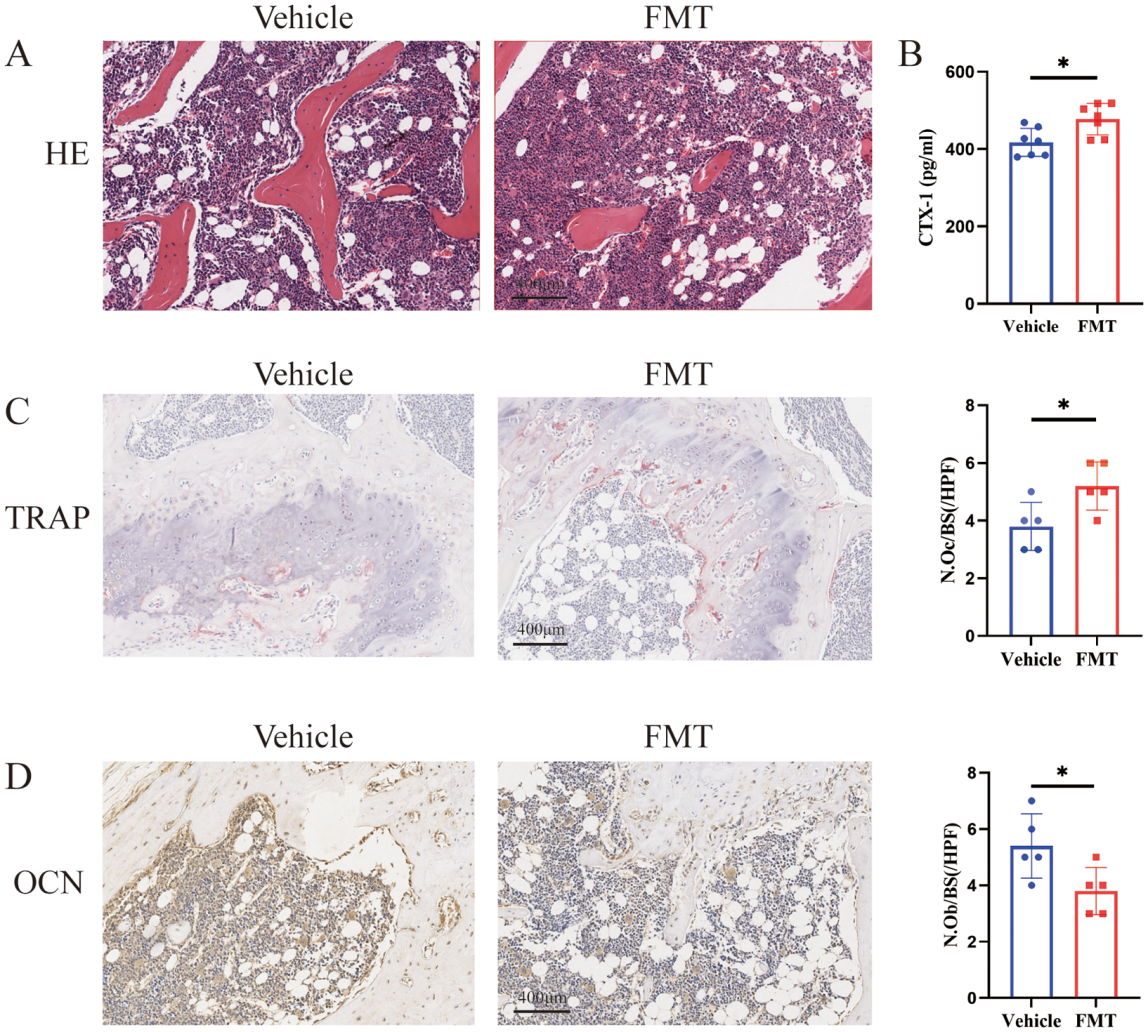
Bone metabolism-related assays and staining. **(A)** Representative H&E stained picture of the proximal femoral region, scale bar 400μm. **(B)** The concentrations of serum CTX-I were detected by ELISA. **(C)** Representative picture and quantification of TRAP staining in the proximal femoral region, red is TRAP staining positive cells. **(D)** Representative picture of OCN staining, brown is osteocalcin positive staining cells. * stands for P<0.05. **(B–D)** Independent-Samples t-test.

The differences in bone mass could be directly reflected by the staining of bone tissue sections. H&E staining of the femur showed a decrease in the area of trabecular bone and trabecular bone itself in the FMT group, indicating obvious bone mass loss (**Figure 3A**). We subsequently examined the concentration of serum CTX-1 in mice and found a significant increase in the FMT group (**Figure 3B**). To further clarify the effect of enterobacteria transplantation on bone mass, Trap staining was performed on femoral samples of mice. Trap staining revealed significant osteoclast differentiation in the FMT group, and the quantitative results of Trap staining indicated a notable increase in the number of osteoclasts along the trabecular bone (**Figure 3C**). At the same time, we analyzed the performance of bone formation in mice. In the analysis of immunohistochemical results, OCN staining of the femur in the FMT group showed a reduction in the number of osteoblasts and attenuated bone formation (**Figure 3D**). These results demonstrated that the bone metabolic status of the mice was changed, as reflected by the enhanced bone resorption and decreased bone formation in the FMT group.

### Gut microbiome profiles of donor population and recipient mice

**Figure 4:**
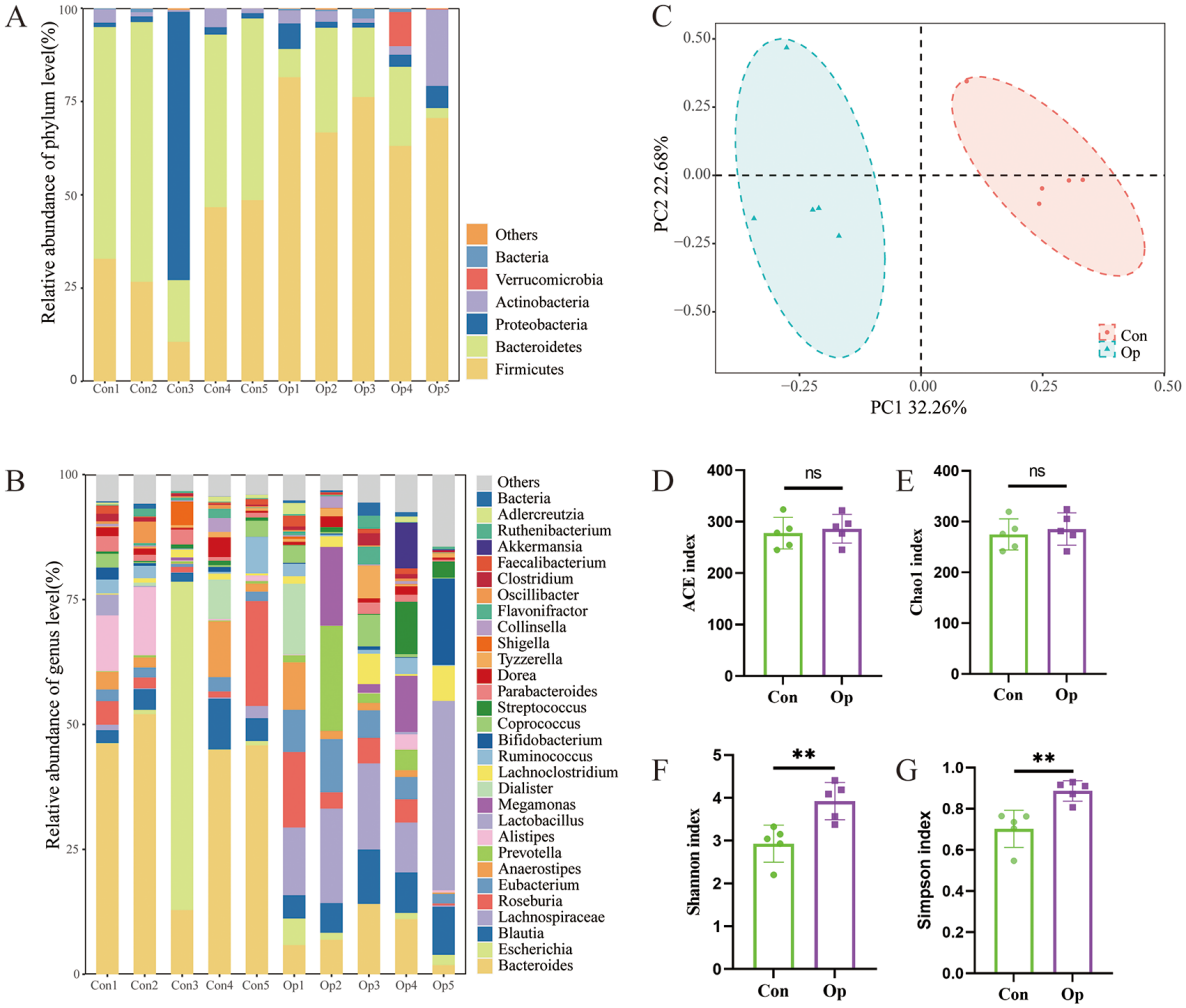
Characteristics of gut microbiota in donor postmenopausal women. **(A)** Stacked bar plots of relative abundance at the phylum level. **(B)** Stacked bar plots of relative abundance at the genus level. **(C)** Principal component analysis. The abscissa represents the first principal component, the ordinate represents the second principal component, and the percentage represents the contribution to the sample variance. Each point represents a sample. **(D-G)** The alpha diversity of intestinal flora in the two groups was described according to ACE index, Chao1 index, Shannon index, and Simpson index. Con, normal bone mass control; Op, postmenopausal osteoporosis group. ns, no statistical difference, ** stands for P<0.01. **(D–G)**, Independent-Samples t-test.

Previous studies demonstrated the important influence of gut microbiota on osteoporosis. We asked about the role of the gut microbiota in altered bone metabolism. To clarify the changes of bacterial flora during FMT. We first used shallow shotgun sequencing to collect data on the donor gut microbiota. Among postmenopausal women, *Firmicutes*, *Bacteroidetes*, *Proteobacteria*, *Actinobacteria*, and *Verrucomicrobes* were dominant phyla (**Figure 4A**). In addition, the relative abundance of the microbiota at genus level was characterized in the two groups of donors, *Bacteroides* (0.40 vs 0.079, p < 0.01) were significantly enriched in the Con group, while *Lachnospiraceae* (0.0080 vs 0.12, p < 0.05) and *Eubacterium* (0.019 vs 0.062, p < 0.05) were significantly enriched in the Op group (**Figure 4B**). To further analyze the differences and similarities of the microbiota between the two groups, Bray-Curtis based principal coordinate analysis (PCOA) was used, and the results showed that the distribution of the microbiota was significantly different between the two groups (**Figure 4C**). Alpha diversity showed that there was no significant difference in ACE index and Chao 1 index between the two groups. The Shannon index and Simpson index of intestinal bacteria in Op group were significantly increased (**Figures 4D–G**). These results showed the gut microbiota increased richness and the significant changes in gut microbiota composition and structure of the Op group.

**Figure 5:**
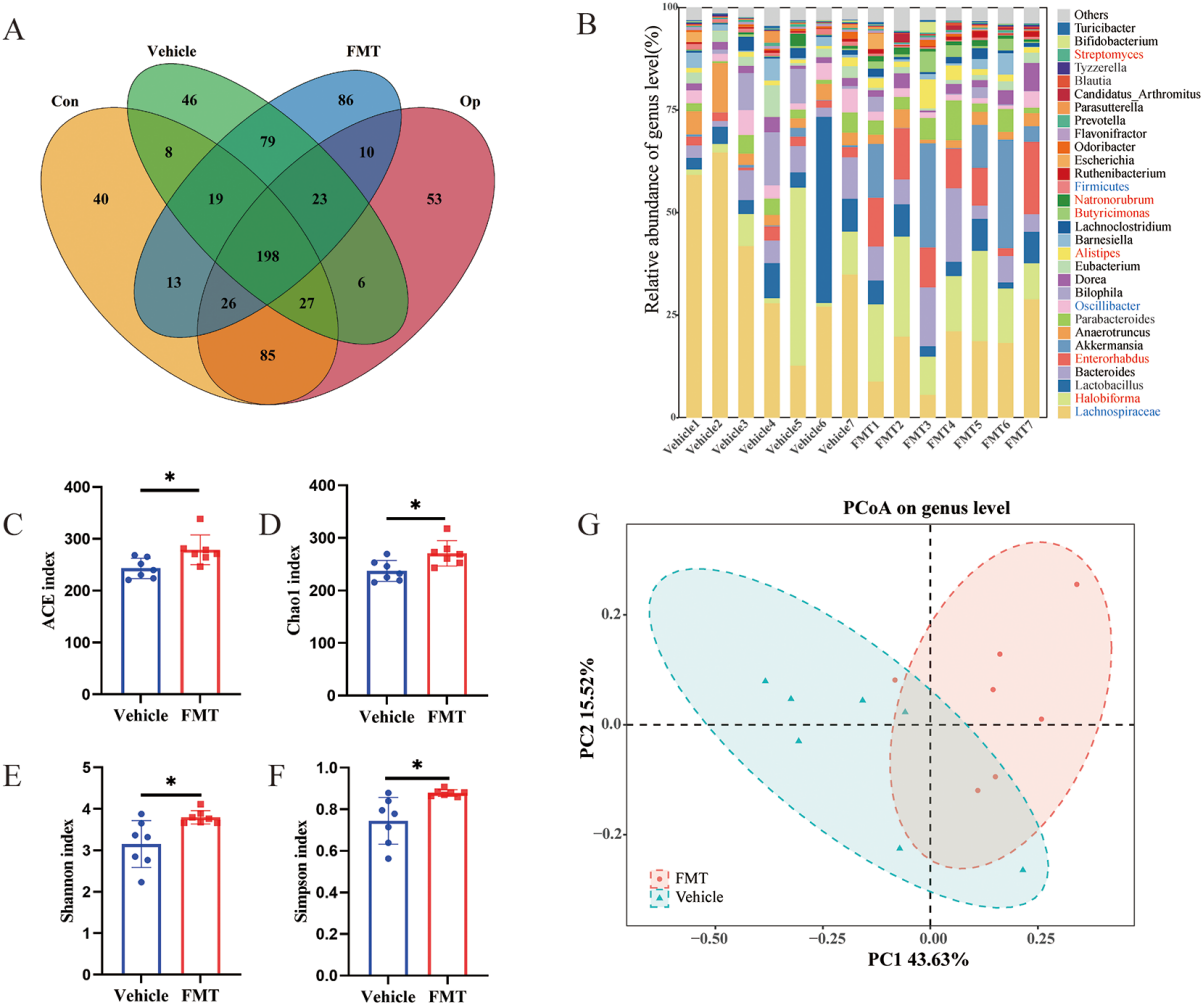
Characteristics of gut microbiota in recipient mice. **(A)** Venn diagram comparing the shared number in gut microbiome of human donors (n = 5 for Con, n = 5 for Op) and recipient mice (n = 7 for Vehicle, n = 7 for FMT) at the genus level. **(B)** Stacked bar plot of relative abundance at the genus level, with a significant difference in red between Vehicle and FMT groups. **(C-F)** Alpha diversity of gut microbiota including ACE index, Chao1 index, Shannon index, and Simpson index. **(G)** The β diversity of the two groups was represented by principal component analysis. * stands for P<0.05. **(C–F)**, Independent-Samples t-test.

To elucidate the colonization of intestinal flora in mice after transplantation, Venn diagram was used to visualize intestinal bacterial colonization. Among the bacterial genera in the FMT group, 257 (60.05%) were consistent with those in the Op group (The total number of genus-level bacteria was 428), and 252 (60.58%) were consistent with those in the Vehicle group (The total number of genus-level bacteria was 416), which showed that the two groups of mice had comparable colonization effect (**Figure 5A**). Similarly, we further analyzed the differences in bacterial abundance at the genus level in mice. We found that among the top 30 bacterial genera in relative abundance, *Lachnospiraceae* (0.38 vs 0.17, p < 0.05), *Oscillibacter* (0.036 vs 0.019, p < 0.05), and *Firmicutes* (0.0093 vs 0.0046, p < 0.05) were significantly increased in the Vehicle group, while *Halobiforma* (0.096 vs 0.16, p < 0.05), *Enterorhabdus* (0.021 vs 0.10, p < 0.05), *Alistipes* (0.010 vs 0.026, p < 0.01), *Butyricimonas* (0.0026 vs 0.020, p < 0.01), *Natronorubrum* (0.0065 vs 0.011, p < 0.05), and *Streptomyces* (0.0020 vs 0.0027, p < 0.05) were significantly increased in the FMT group (**Figure 5B**). To further clarify whether the changes in microbiota richness in human subjects were successfully replicated in mice, we analyzed the alpha diversity of the two groups of mice. The results showed that ACE index, Chao 1 index, Shannon index, and Simpson index were significantly increased in the FMT group (**Figures 5C–F**). PCOA analysis also showed a significant difference in the microbiota composition between the two mice groups (**Figure 5D**). These results indicated that the composition and structure of gut microbiota in FMT mice were altered similarly to those in Op mice, as reflected by the increased richness of microbiota and the significant increase in the relative abundance of specific bacterial groups.

### The changes of bacterial flora at genus and species levels reveal the characteristic changes of bacterial flora in mice after FMT

**Figure 6:**
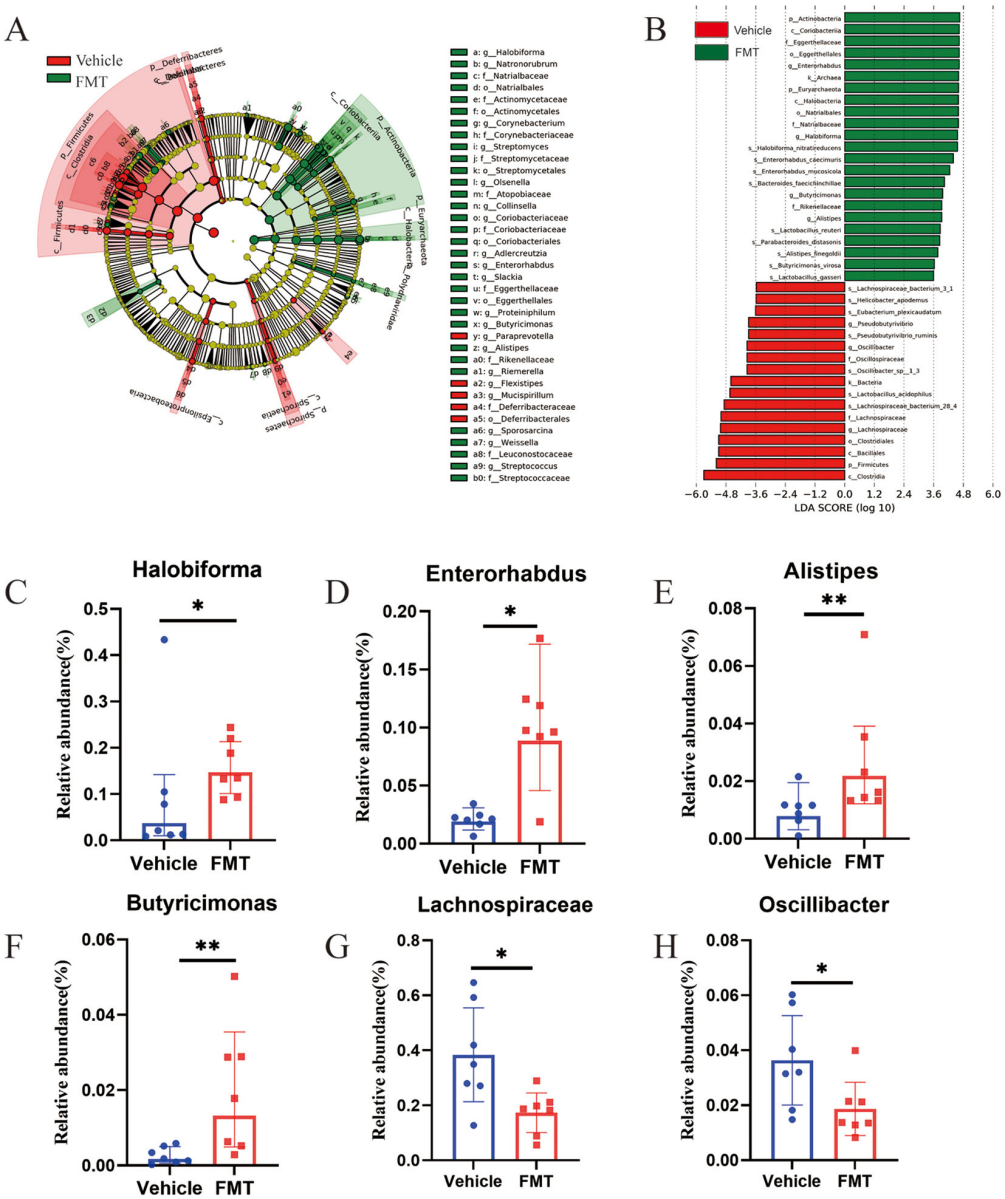
Analysis of differences in bacterial flora structure at the genus level in mice. **(A)** The Least discriminant analysis (LDA) effect size taxonomic cladogram, with radiation circles from inside to outside indicating taxonomic level from phylum to species. Each small circle represents a classification at that level, and the diameter of the small circle is proportional to the corresponding relative abundance. Where yellow is the species with no significant difference, red nodes represent the microbiome that plays an important role in the Vehicle group. Green nodes represent the microbiome that plays an important role in the FMT group. The species names represented by the English letters in the figure are shown on the right. **(B)** Histogram of LDA value distribution showing statistical differences (LDA score>3.5). The histogram length indicates the effect of different species (LDA score). **(C-H)** At the genus level, six dominant bacteria were significantly enriched in the FMT group with statistical differences and biological significance. * stands for P<0.05, and ** stands for P<0.01. **(B–H)**, Kruskal-Wallis test and paired Wilcoxon rank-sum test.

To further identify the intestinal flora affecting bone mass metabolism. LEfSe analysis was employed to identify statistically and biologically significant species. The cladogram revealed significantly enriched genera, seventeen bacterial genera were enriched in the FMT group and 3 bacterial genera were enriched in the Vehicle group (**Figure 6A**). To screen out the final differential bacteria (biomarker), we set the LDA value to >3.5. The enriched bacterial genera were visualized as LDA histograms. Finally, we identified 4 dominant genera that were significantly enriched in the FMT group, including *Halobiforma* (0.096 vs 0.16, p < 0.05), *Enterorhabdus* (0.021 vs 0.10, p < 0.05), *Alistipes* (0.010 vs 0.026, p < 0.01), *Butyricimonas* (0.0026 vs 0.020, p < 0.01). Besides, two dominant genera in the Vehicle group, including *Lachnospiraceae* (0.38 vs 0.17, p < 0.05), *Oscillibacter* (0.036 vs 0.019, p < 0.05) (**Figures 6B–H**).

### Changes in intestinal permeability

**Figure 7:**
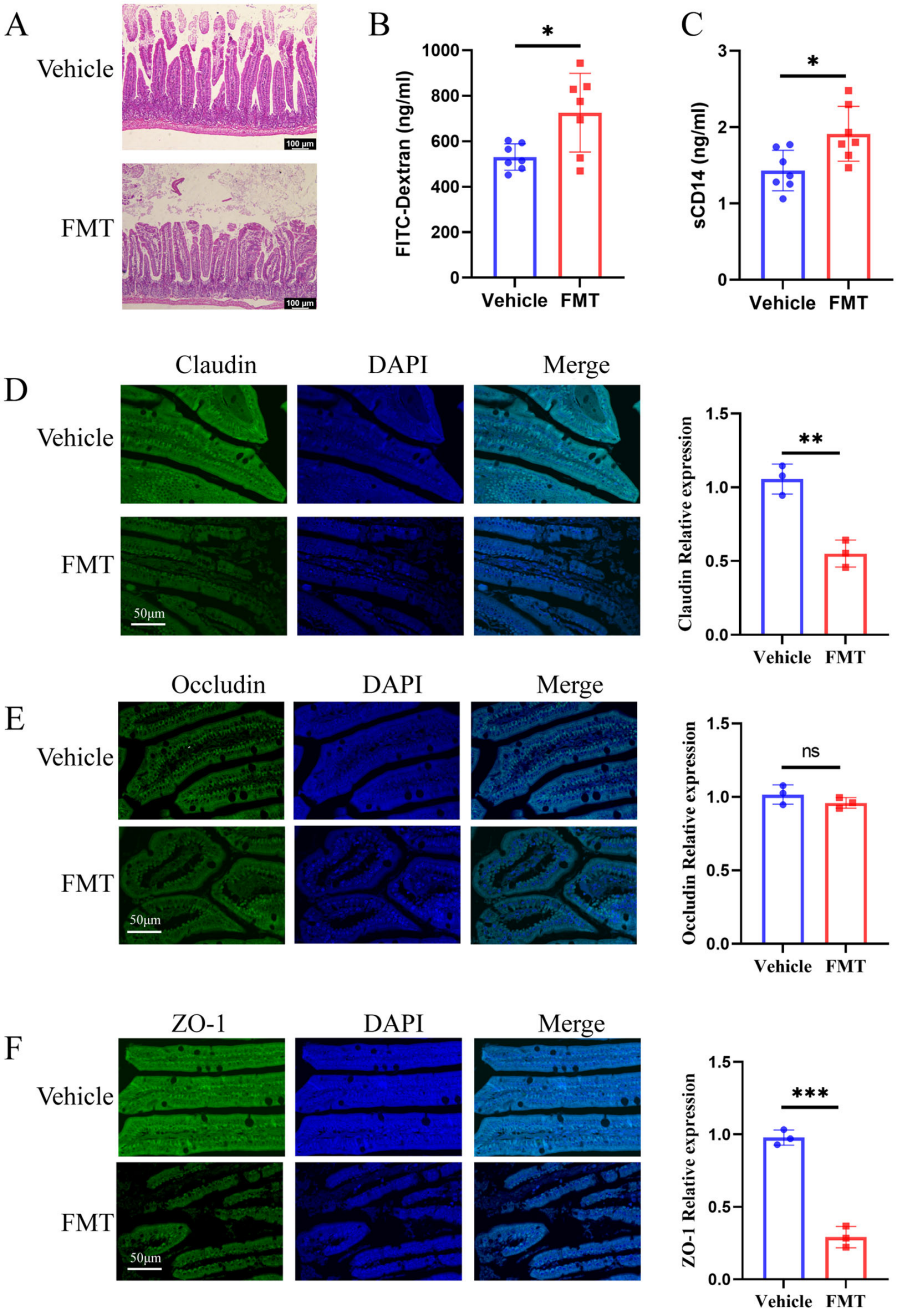
Recipient mice showed increased intestinal permeability. **(A)** H&E staining of ileal tissues. In the FMT group, the villi were arranged disorderly, swelling was obvious, and the intestinal epithelial barrier was damaged. **(B, C)** The concentrations of serum LPS and sCD 14 were detected by ELISA. **(D-F)** The Claudin, Occludin, and ZO-1 immunohistochemical staining and quantification of ileum tissue sections. The scale bar was 50μm. * stands for P<0.05, ** stands for P<0.01, and *** stands for P<0.001. **(B-F)**, Independent-Samples t-test.

The underlying mechanisms of bone loss induced by these dominant species were further explored. Our previous work had demonstrated the critical role of gut barrier impairment in the gut microbiota-osteoporosis axis (Wang et al., 2022a). We therefore first asked whether gut microbiota derived from human donors could contribute to gut barrier damage in mice. H&E staining showed obvious intestinal mucosa damage in the FMT group, with aggravated swelling and disordered villus structure (**Figure 7A**). In line with these observations, FMT group mice showed increased small intestine permeability for FITC-dextran MW 4000 (FD4) (**Figure 7B**). To add more evidence of intestinal barrier dysfunction, we analyzed serum sCD14 levels, which has been described as a marker of microbial translocation. ELISA results showed that the serum sCD14 content of mice in the FMT group was significantly increased (**Figure 7C**). To obtain further direct evidence of intestinal permeability impairment, immunohistochemical staining was used to detect the content of Claudin, Occludin, and ZO-1 related to intestinal permeability. Immunohistochemical staining revealed significantly reduced expression of claudin, occludin, and ZO-1, which were related to the integrity of the intestinal epithelial barrier, compared with the Vehicle group. Staining quantification demonstrated statistically significant differences in the expression of claudin and ZO-1 between the two groups (**Figures 7D-F**). These results demonstrated significantly damage to the intestinal barrier in mice transplanted with gut bacteria from osteoporosis patients.

### Bone loss and impaired intestinal barrier were significantly correlated with gut microbiota

**Figure 8:**
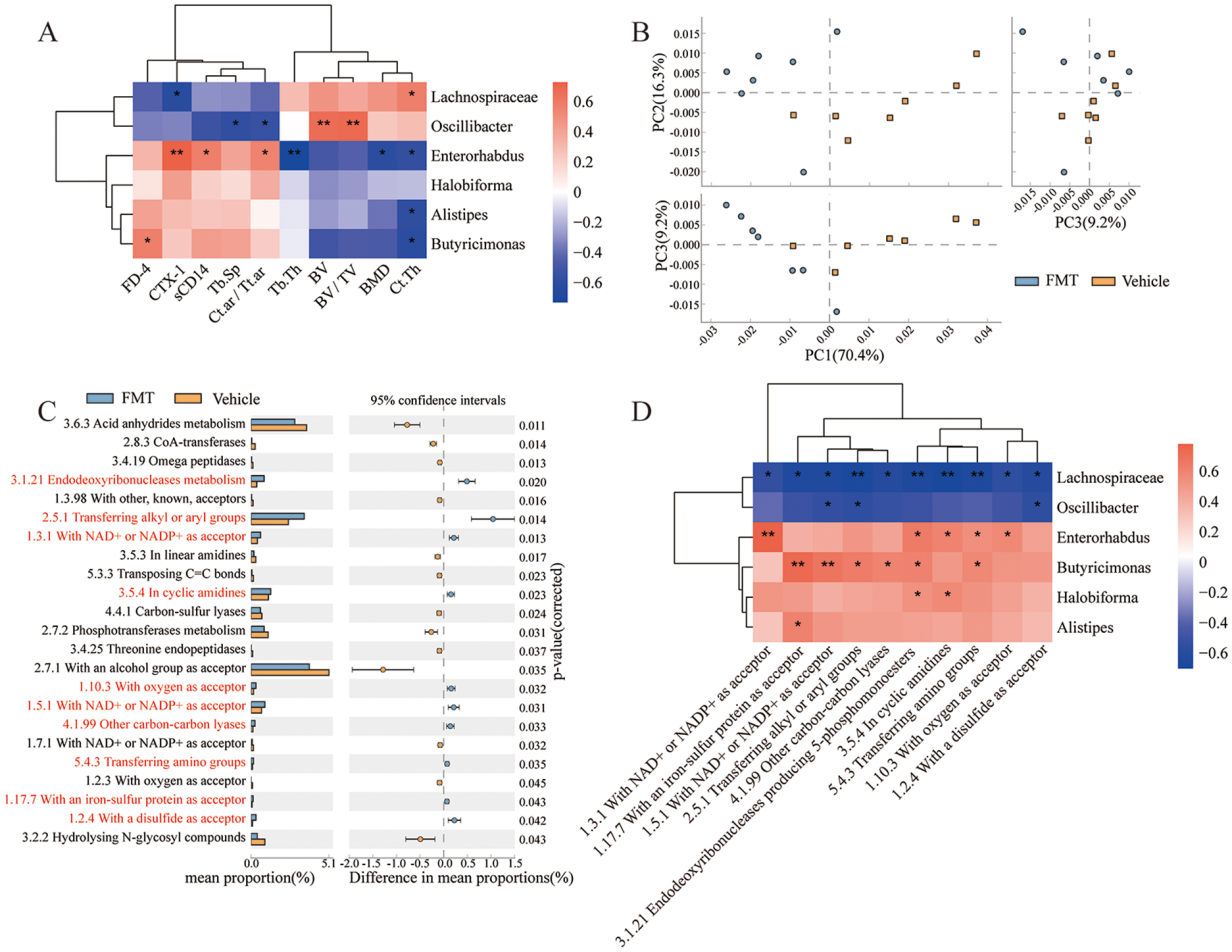
Functional analysis and correlation analysis. **(A)** Correlation between dominant bacteria and bone tissue parameters. **(B)** PCA plot, comparing the classification profiles of enzymes in all samples of the two groups of mice. **(C)** Differentially expressed enzymes between the two groups by STAMP analysis. Each bar on the left represents the mean value of each enriched enzymes in each group with significant differences. Differences between the confidence levels of the groups are shown on the right. The two ends of each circle in the figure indicate the upper and lower 95% confidence intervals of the mean differences. The center of the circle indicates the difference in the means. (Corrected P value < 0.05 is defined as a significant difference. Orange, Vehicle; Blue, FMT. Red indicates significantly enriched enzymes in the FMT group.). **(D)** Correlation between dominant bacteria and enriched pathways. * stands for P<0.05, and ** stands for P<0.01. Correlations analysis were performed by Spearman Analysis.

To clarify the role of gut microbiota disorder in bone loss and intestinal barrier damage, the correlation of bone loss and impaired intestinal barrier with gut microbiota was visualized as heatmaps based on Spearman analysis. We found a significant positive correlation between *Lachnospiraceae* and Ct.Th, and a significant negative correlation between *Lachnospiraceae* and CTX-1. *Oscillibacter* was significantly positively correlated with BV and BV/TV, and negatively correlated with Tb.Sp, Ct.ar/Tt.ar. *Enterorhabdus* was negatively correlated with BMD, Tb.Th, and Ct.Th, and positively correlated with Ct.ar/Tt.ar, sCD14, and CTX-1. *Alistipes* was significantly negatively correlated with Ct.Th. *Butyricimonas* was negatively correlated with Ct.Th and positively correlated with FD-4 (**Figure 8A**). These results suggested that bone loss and gut barrier impairment are significantly associated with dysbiosis.

### The enzyme expression profile of recipient mice was changed and related to intestinal bacteria

To investigate the effect of gut microbiota on the expression function of specific genes, we utilized PICRUST to predict the function of sequencing data based on the integrated relational enzyme database IntEnz. This approach helped identify biologically relevant differences between the two groups. Principal coordinate analysis revealed significant differences in the enzyme expression functions of the two groups under the three principal components (**Figure 8B**). Expanded error bar plots were used to characterize the differentially expressed enzymes between the two groups, and 10 enzymes were significantly up-regulated. In contrast, 13 enzymes were significantly down-regulated in the FMT group (**Figure 8C**). Furthermore, Spearman correlation analysis based on heatmap was performed to determine the correlation between the differentially expressed enzymes and the gut microbiota (**Figure 8D**). Overall, *Lachnospiraceae* was strongly associated with the detected enzymes whose expression were significantly upregulated in the FMT group. *Enterorhabdus* and *Butyricimonas* also played important roles in regulating the characteristic enzyme profile. In addition, *Oscillibacter* interacts with the enzyme with a disulfide as acceptor (1.2.4), and there was a significant negative correlation between the expression of enzymes with NAD+ or NADP+ as acceptor (1.5.1) and enzymes transferring alkyl or aryl groups (2.5.1). *Halobiforma* was positively correlated with Endodeoxyribonucleases producing 5-phosphomonoesters (3.1.21) and the enzymes in cyclic amidines (3.5.4). *Alistipes* were positively correlated with the enzymes with an iron-sulfur protein as acceptor (1.17.7). These results suggest that the transplanted gut microbiota altered the expressed enzyme profile of the mice, which has important implications for the regulation of metabolic reactions in which the enzymes are involved.

### Biochemical changes in blood and bone after microbiota transplantation

**Figure 9:**
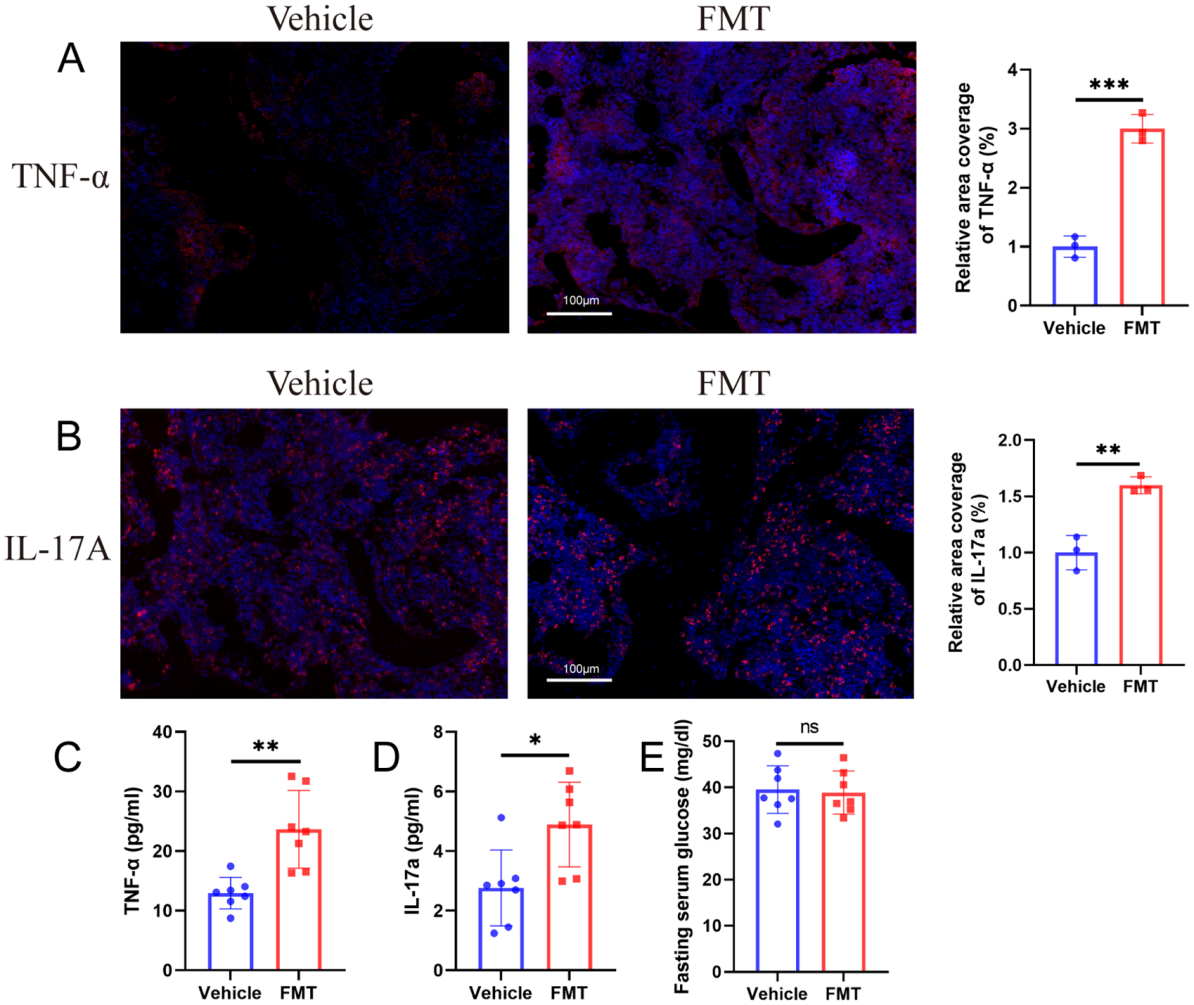
Biochemical changes in blood and bone after microbiota transplantation. **(A, B)** Immunofluorescence staining and quantification of TNF-α and IL-17A in the proximal femur of mice. TNF-α+ or IL-17A+ cells are shown in red, nuclei are shown in blue DAPI, scale bars 100 μm. **(C, D)** ELISA for mice serum TNF-α and IL-17A. **(E)** Fasting blood glucose of mice. ** stands for P<0.01, and *** stands for P<0.001. **(B–E)**, Independent-Samples t-test.

To explore whether the bacterial translocation caused inflammation after the change of intestinal barrier after transplantation, we used immunofluorescence to detect the expression of TNF-α and IL-17A in the proximal femur of mice. The immunofluorescence results showed that the inflammatory markers (TNF-α and IL-17A) in the femur of mice in the FMT group were significantly up-regulated. Quantitative results showed that expression levels were significantly different between the two groups (**Figures 9A, B**).

Bacterial translocation and femoral inflammation are usually accompanied by inflammatory changes in the blood of mice, which may be the connection mechanism of intestinal barrier and bone mass loss. Therefore, we detected the changes of TNF-α and IL-17A in the serum of mice, and the results showed that the serum TNF-α and IL-17A in the FMT group were significantly increased (**Figures 9C, D**).

In addition, the fasting blood glucose content of the two groups of mice was examined, and the results showed no statistically significant difference in blood glucose between the two groups of mice (**Figure 9E**), which ruled out impaired glucose tolerance after fecal microbiota transplantation.

## Discussion

The pathogenic role of intestinal flora has been reported. In patients with hypertension, the dysregulated intestinal flora produces excessive lipopolysaccharide (LPS), which was considered to be directly related to the occurrence of hypertension (Li et al., 2017), Endotoxin-producing bacteria transplanted into germ-free mice induced obesity and insulin resistance (Le Roy et al., 2013). Here, we demonstrated the following findings: (1) Transplantation of gut microbiota from postmenopausal osteoporosis patients into pseudo-sterile mice resulted in accelerated bone loss; (2) Intestinal microbiota transplantation from postmenopausal osteoporosis patients to pseudo-sterile mice increased intestinal permeability, caused intestinal mucosa damage, and reduced the expression of tight junction proteins ZO-1 and claudin in the intestinal barrier; (3) Changes in bone metabolism, intestinal permeability and specific enzyme functions were associated with changes in the abundance of *Lachnospiraceae*, *Enterorhabdus*, *Butyricimonas*, *Oscillibacter*, *Alistipes*, and *Halobiforma*. (4) The inflammation of blood and bone caused by bacterial translocation after intestinal barrier damage may be a potential connection mechanism between the gut and bone. These findings suggested that these bacteria may be associated with postmenopausal osteoporosis, contributing to accelerated bone mass loss in pseudo-sterile mice.

We obtained pseudo-sterile mice using ABX treatment, which led to significant changes in the diversity and composition of the host gut microbiota (Becattini et al., 2016). While host mice did not eliminate bacteria after ABX treatment, colony counting before and after ABX treatment indicated a significant reduction in bacterial load, leading to changes in signaling pathways, organ morphology, and cell population, similar to germ-free mice (Kennedy et al., 2018), therefore, the results of ABX treatment were considered to be acceptable. Specifically, in this study, differences in gut microbiota distribution between the donors and recipient groups coincided with significant differences in bone mass changes between the corresponding FMT and vehicle groups. However, certain human-specific bacteria, such as *Mitsuokella*, *Megasphaera*, and *Dialister*, failed to colonize mice due to differences in diet, environment, and gut characteristics (Nguyen et al., 2015).

We found alterations in body weight after transplantation of bacteria from postmenopausal osteoporotic mice. Some previous articles related to fecal microbiota transplantation in disease models did not mention the weight change of mice (Kelly et al., 2016; Li et al., 2017; Kim et al., 2021), which proved that the weight change caused by fecal microbiota transplantation was not universal. Body weight changes in postmenopausal osteoporosis models had been reported in previous work (Zhang et al., 2022; Huang et al., 2024), so we believe that the body weight changes in the mice in the present study were specific. We examined the fasting blood glucose content in the serum of the mice and showed that there was no significant difference in fasting blood glucose between the two groups of mice, preliminarily excluding the problem of insulin function in the mice. One of the causes of weight change in postmenopausal osteoporosis was abnormal lipid metabolism (Huang et al., 2024). Microbiota transplantation transferred some of the features of postmenopausal osteoporosis, so we thought that weight gain after microbiota transplantation might be due to increased fat mass in the mice.

To assess bone metabolic status, we performed Micro-CT and obtained bone tissue parameters for the two groups of mice. Previous studies had demonstrated that mice with bone mass loss exhibit significant damage to the bone microstructure, with an enlarged medullary cavity and thinner cortical bone (Liu et al., 2021). Our three-dimensional reconstruction of the FMT group was consistent with these findings. We further confirmed statistical differences in bone tissue parameters between the two groups. BMD was generally regarded as a predictor of fracture risk in clinical practice (Barron et al., 2020). The significant decrease in BMD, BV, and BV/TV in mice receiving osteoporotic enterobacteria transplantation indicated that the mice in the FMT group had substantial bone loss. Reduced trabecular thickness and increased trabecular separation were significant features of osteoporosis (Akhter et al., 2007). This was consistent with our study. Combined with serum CTX-1, HE, and histochemistry, we believed that the characterization of accelerated bone loss in postmenopausal osteoporosis patients was successfully transferred to mice.

Postmenopausal osteoporosis was a bone metabolic disease characterized by estrogen deficiency, and previous studies had highlighted the critical role of gut microbiota in bone loss in estrogen-deficient osteoporotic mice (Guan et al., 2023). Estrogen deficiency could lead to the impairment of the intestinal barrier, which in turn leaded to the translocation of gut microbiota, activated the immune response, and ultimately leaded to increased bone resorption (Xu et al., 2017). Epithelial tight junction proteins, including Claudin, Zo-1, and Occludin, were important components of the intestinal barrier (Turner, 2009). In this study, the FMT group showed significant down-regulation of claudin and zo-1 after gut microbiota inoculation, and H&E suggested the appearance of swelling disorder of the intestinal mucosa, indicating impaired intestinal barrier function. The increased intestinal permeability was also confirmed by content of serum FITC-dextran (FD4) and sCD14. Our previous work demonstrated the role of gut microbiota in gut barrier repair by transplanting microbiota from young rats into aged rats (Wang et al., 2022a). Therefore, we hypothesized that gut microbiota dysbiosis was an important factor of gut barrier impairment.

Previous studies had shown that damage to the intestinal epithelial barrier can lead to the translocation of pathogens into the blood, causing inflammatory manifestations (König et al., 2016; Nagao-Kitamoto et al., 2016). The connection between intestinal barrier changes and bone loss after intestinal microbiota transplantation in mice may be achieved through specific inflammatory cells and inflammatory factors. The direct link between intestinal disorders and bone metabolism has been reported, and the gut-immune bone axis was a convincing way for intestinal bacteria to regulate bone metabolism (Lyu et al., 2023; Li et al., 2024). Intestinal flora regulates the balance between Th17 and Treg cells, which was one of the important ways to affect bone metabolism. Th17 cells secrete increased proinflammatory factors (IL-17A, TNF-α), which promoted osteoclast differentiation and lead to increased bone resorption. In contrast, Treg cells inhibit osteoclast formation and promoted bone formation by secreting anti-inflammatory cytokines such as IL-4, IL-10, and TGF-β (Guo et al., 2023). Studies had reported an increase in the content of specific T cells in the peripheral blood of ovariectomized (OVX) mice (Adeel et al., 2013). However, increased TNF-α in osteoporosis patients had been reported (D’Amelio et al., 2008). In our study, we detected an up-regulation of blood TNF-α expression in the FMT group, which was consistent with the occurrence of inflammation after FMT transplantation. The increase of TNF-α mediated by Th17 TNF-α^+^ T cells was considered to be an important factor in bone loss, and the upregulation of CCL20 expression guided the increased Th17 cells (CD4^+^ IL-17A^+^ T cells) in the gut to migrate to the bone marrow (Yu et al., 2020). Yu et al. further elucidated the connection mechanism. They clarified the upregulation of gut Th17 and TNF-α^+^ T cells in a model of postmenopausal osteoporosis and highlighted that OVX increased their S1P receptor 1-mediated (S1PR1-mediated) outflow from the gut and enhanced their subsequent influx into bone marrow via CXCR3 and CCL20 mediated (Yu et al., 2021). We examined inflammatory changes in mice femurs and found that the expression of IL-17A and TNF-α were upregulated after microbiota transplantation. This was consistent with previous findings. Therefore, we suggested that one possible mechanism of bone mass change after gut microbiota transplantation is inflammation caused by microbiota translocation, thereby activating the gut-immune-bone axis. However, the deeper mechanisms of the intestinal bone junction still needed to be further studied.

The diversity analysis of the two groups of donors showed that the diversity of the postmenopausal osteoporosis group increased, and the difference was significant compared with the Con group. After fecal microbiota transplantation, we examined the alpha diversity of the mice and found that the mice receiving the fecal microbiota from the Op patients showed the same changes in alpha diversity as those from the donors. β diversity analysis also revealed significant differences in the composition and structure of the microbiota between the two groups of mice, this suggests that FMT successfully transferred the characterization of gut microbiota dysbiosis in osteoporosis patients. A growing body of evidence suggests that gut microbiota disturbance is objectively present in postmenopausal osteoporosis.

*Lactobacillus reuteri* supplementation can reduce intestinal permeability and alleviate bone loss in OVX mice (Collins et al., 2016). This supported the view that specific gut microbiota may play a key role in gut barrier regulation and bone metabolism. Therefore, genus-level analysis was performed to identify the dominant gut microbiota. In previous studies on gut microbiota and osteoporosis, the cutoff for revealing genus-level microbiota characteristics was inconsistent. Wang et al. selected 21 as the cut-off value at the genus level (Wang et al., 2017), and Xu et al. selected 10 (Xu et al., 2020). We optimized for this. Genera with relative abundance above 30 accounted for less than 10% of the total population. We considered the top 30 genera to be the ideal cut-off value. Based on this, six dominant genera were identified and their correlations with bone tissue parameters and intestinal permeability indexes were analyzed our results of correlation analysis of bone tissue parameters and intestinal barrier permeability with the final six dominant genera identified in mice reinforce this view.

In this study, we clarified the significant enrichment of *Lachnospiraceae* in the control group. *Lachnospiraceae* was one of the bacteria with high relative abundance in human gut, which played an important role in maintaining host health. Reduced *Lachnospiraceae* content had been observed in a variety of diseases, including colorectal cancer, type 2 diabetes, and inflammatory bowel disease (Vacca et al., 2020; Zhang et al., 2023). Recent findings also provided direct evidence of skeletal augmentation in Lachnospiraceae (Wang et al., 2024). In addition, the correlation between bone metabolism and intestinal permeability suggested the potential of *Lachnospiraceae* in the fight against bone loss. Interfering with the composition of *Lachnospiraceae* in the gut may be a potential approach to regulate bone metabolism.

On the other hand, we identified some enzymes significantly enriched in the experimental group based on their functions, which included oxidation-reduction reactions, hydrolysis reactions, cleavage reactions, and isomerization reactions in mice. These enzymes were involved in the extensive regulation of intestinal microbes in the body. Previous studies had suggested that the influence of intestinal bacteria on bone metabolism was a multi-system and complex regulatory process (Hernandez et al., 2016), which was consistent with our study. We found a significant positive correlation between *Alistipes* and enzymes with iron-sulfur protein as an acceptor. Iron-sulfur proteins played a fundamental role in mitochondria, assisting many essential cell biological processes (Lill and Freibert, 2020). They were closely related to the maturation and realization of complete functions of mitochondria, and mitochondrial homeostasis played a pivotal role in the regulation of bone resorption and bone formation (Lee et al., 2021; Das et al., 2022). This could be one of the potential mechanisms by which *Alistipes* affects bone metabolism.

This study has several limitations. First, our study supported a causal relationship between these bacteria and osteoporosis, but the sample size of the human study was relatively small. Therefore, our results may reflect only the specific population tested. A larger cohort of postmenopausal osteoporosis patients and single bacterial colonization with potentially pathogenic bacteria are needed to further elucidate the specific effects of the gut microbiome on osteoporosis. Secondly, although we observed changes in the inflammatory state after microbiota transplantation, we were unable to further perform multi-omics analysis to understand the underlying mechanisms.

## Conclusion

Overall, our findings indicated that gut microbiota from osteoporosis patients accelerates bone mass loss in mice, aberrant gut microbiota may play a causal role in the development of bone mass loss by disrupting the gut barrier. Based on the analysis of gut microbiota, *Lachnospiraceae*, *Enterorhabdus*, *Butyricimonas*, *Oscillibacter*, *Alistipes*, and *Halobiforma* may be an important bacterium regulating the bone metabolism and intestinal barrier. Blood and bone inflammation caused by bacterial translocation after intestinal barrier injury may be responsible for bone mass loss. We provided important evidence for a novel role of gut microbiota dysbiosis as a change in bone mass.

## Data Availability

The original contributions presented in the study are publicly available. This data can be found here: [https://www.ncbi.nlm.nih.gov/, PRJNA1192539].
